# Increased acromiohumeral distance in a double-row arthroscopic rotator cuff surgery compared to a single-row surgery after 12 months

**DOI:** 10.1186/s13018-021-02523-1

**Published:** 2021-06-16

**Authors:** Kaya Turan, Haluk Çabuk, Cenk Köroğlu, Çağatay Öztürk

**Affiliations:** 1 Department of Orthopedics and Traumatology, Medicine Faculty of Istinye University, Aşık Veysel Mah. No:1 Istinye University Liv Hospital Esenyurt, İstanbul, Turkey; 2 Department of Orthopaedics and Traumatology, Tekirdag Ismail Fehmi Cumalıoglu City Hospital, Eski Cami Mah, Hastane Sk. No:1 Suleymanpasa, Tekirdağ, Turkey

**Keywords:** Acromiohumeral distance, Arthroscopy, Double-row, Rotator cuff repair, Integrity, Single-row

## Abstract

**Background:**

Arthroscopic rotator cuff surgery is an effective treatment for rotator cuff tears with the considered use of double-row repair techniques becoming popular in the last decade. We aim to compare the effects of double- and single-row arthroscopic rotator cuff repairs (ARCR) on repair integrity (RI) and acromiohumeral distance (AHD).

**Methods:**

In this observational study, we retrospectively identified 98 patients with degenerative rotator cuff tear treated with arthroscopic rotator cuff repair between 2016 and 2019. We excluded 22 patients with partial-thickness tears, 15 with associated subscapularis or SLAP tears, 13 with massive tears, and 5 patients lost to follow-up; we included 43 patients who had ARCR for full-thickness cuff tear and clinical, radiologic follow-up. Of these 43 patients, 23 are grouped as double-row repair group (DRG) and 20 as single-row repair group (SRG). A minimum of 12 months after the surgery, bilateral shoulder MRIs were obtained. Contralateral shoulders without asymptomatic rotator cuff tears served as a control group (CG). The operating surgeon and two other surgeons experienced in arthroscopy blindly measured the AHD and determined the RI at the control MRIs in all groups. Functional assessments relied on UCLA and qDASH Scores.

**Results:**

The mean age was 57.89 (45–78) years, and the mean follow-up time was 28,65 (21–43) months. The mean AHD of the CG was 9.7 ± 0.96 mm, the preoperative AHD of DRG was 8.62 ± 1.45 mm, and SRG was 9.71 ± 0.95 mm. The postoperative mean AHD of DRG 9.61 ± 1.83 mm and SRG was 10.21 ± 1.97 mm. AHD differences between the preoperative and postoperative groups were significant (*P=0.009*). The increase of the AHD in the double-row group was significantly higher than the single-row group (*P=0.004*). There was a high correlation between the RI and DASH scores (*P=0.005*). RI did not correlate with the repair method (*P=0.580*).

**Conclusion:**

Although double-row repairs can maintain greater AHD than single-row repairs in the clinical setting, this difference did not affect functional results. Regardless of the surgical intervention, functional results are favourable if RI is achieved.

**Level of evidence:**

Level III, Retrospective Cohort Study

## Introduction

Over the last two decades, surgeons have come to choose arthroscopic methods over open surgical procedures. Arthroscopic rotator cuff surgery causes minimal injury to healthy tissues and preserves unique anatomy more than the open approach. Healing after arthroscopic rotator cuff surgery is faster, and the recovery period is shorter. Arthroscopic rotator cuff surgery provides satisfactory results for selected patients with degenerative tears [1–5]. Various arthroscopic repairing techniques have been historically described [6–8], with the considered use of double-row repair techniques becoming popular in the last decade. Although it has been suggested that double-row sutures are more durable and related to reduced rates of re-rupture and superior clinical outcomes than single-row repairs, the most recent research has shown that there is no significant difference between the clinical outcomes and re-tear rates of the two repair methods [2, 4, 8]. Another important parameter is acromiohumeral distance. The relationship between rotator cuff tears and AHD is such that as AHD decreases, the incidence of a tear increase s[9–13]. We hypothesized that with greater AHD and subacromial volume, we can decrease re-rupture rates and gain better long-term clinical outcomes. Our observational study aimed to document the comparable effect of the surgical method on repair integrity (RI) and acromiohumeral distance (AHD).

## Materials and methods

We retrospectively identified patients with degenerative rotator cuff tear treated with arthroscopic rotator cuff repair and acromioplasty between the years 2016 and 2019 at a tertiary training and research hospital; ethical approval was waived by a local ethics committee in view of the retrospective nature of the study. We conducted our study in accordance with the principals of the Helsinki Declaration. Our study included patients with repairable rotator cuff tear diagnosed clinically and observed on magnetic resonance imaging, who had symptoms for at least 3 months that were unresponsive to nonoperative management. Our study included small to medium sized repairable full-thickness tears and excluded associated subscapularis or SLAP tears, grade 3–4 fatty infiltration (Goutallier Classification), acute traumatic tears, shoulder instability, acromioclavicular and glenohumeral arthritis, inflammatory arthritis, history of previous shoulder surgery, labral lesion, and adhesive capsulitis. All operations were performed by one surgeon (KT) in beach chair position with the routine arthroscopic portals. The tear size was measured with a ruler on the probe. Regardless of the tear type, a single-row repair was applied to the first 20 patients and double-row for next 23 patients. A limited anterolateral acromioplasty was routinely performed in all patients. Biceps tenotomy was performed if there is an associated biceps pathology. For single-row repair, one suture anchor was used and for double-row repair, and two suture anchors were used. A sling without abduction pillow was applied, and a passive range of motion exercises was initiated on the day after surgery. The rehabilitation protocol for all patients was continued under the supervision of the operating surgeon. The patients were allowed only passive range of motion until 6th week. Then, the sling was removed, and a gradual active range of motion exercises started. The rehabilitation process continued with progressive strengthening by an experienced shoulder physiotherapist for a total of 3–4 months. Bilateral shoulder MRIs were obtained at least 12 months after surgery. Functional assessments were done during final follow-up, using the University of California at Los Angeles (UCLA) Shoulder and the Quick Disabilities of the Arm, Shoulder, and Hand (qDASH) Scores.

**Fig. 1:**
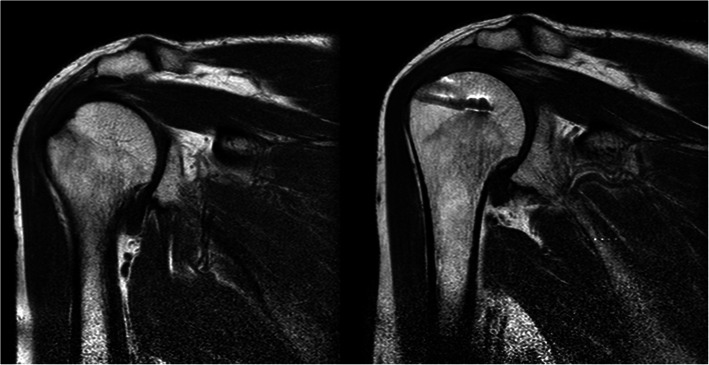
Measurement of acromiohumeral distance (AHD)

**Fig. 2:**
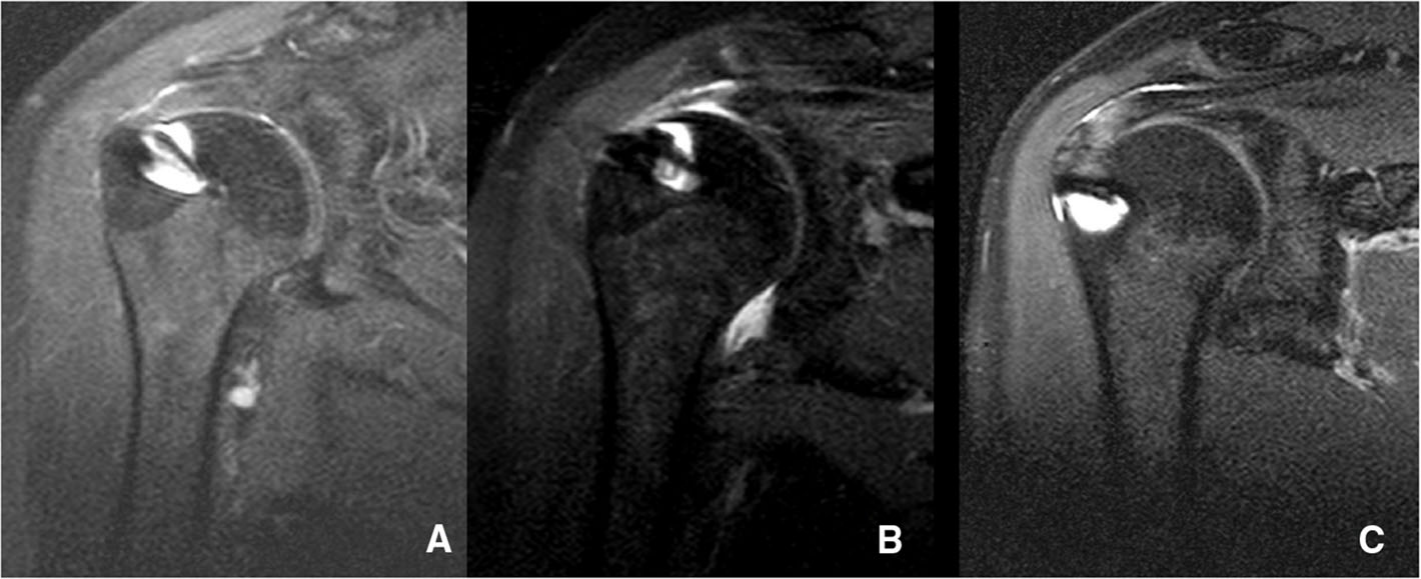
Assessment of repair integrity on T2-weighted MRI

The operating surgeon and two surgeons experienced in arthroscopy blindly measured the AHDs on the T1-weighted MR images of the preoperative, postoperative, and contralateral shoulder MRIs. The distances are measured as Saupe et al. [9] described. A line is drawn from lower surface of acromion to center of humeral head and the shortest distance from the highest level of humeral head to acromion is measured on a dicom software (Fig. 1). The RI was also assessed on the postoperative MRI, according to parameters described by Sugaya et al. [14]. They defined five groups for determining RI: Type 1 is described as enough thickness with homogenously low intensity; type 2, enough thickness with partial high intensity; type 3, insufficient thickness without discontinuity (thinned cuff); type 4, presence of minor discontinuity; and type 5, the presence of a significant discontinuity (Fig. 2). The ratio of asymptomatic rotator cuff tear at the contralateral side was 53%. The statistical analyses were performed by SPSS version 25 for Windows 10. The interobserver reliability for distance measurements, and RI classifications were evaluated by intraclass correlation coefficient (ICC). An independent sample t test was used to compare preoperative, postoperative, and control group AHDs. The correlation between the changes in the AHDs and the functional scores was analyzed using Pearson Correlation analysis. *P values* smaller than 0.05 were considered as statistically significant. Our study was reviewed and approved by the ethics committee of a local university.

## Results

**Fig. 3:**
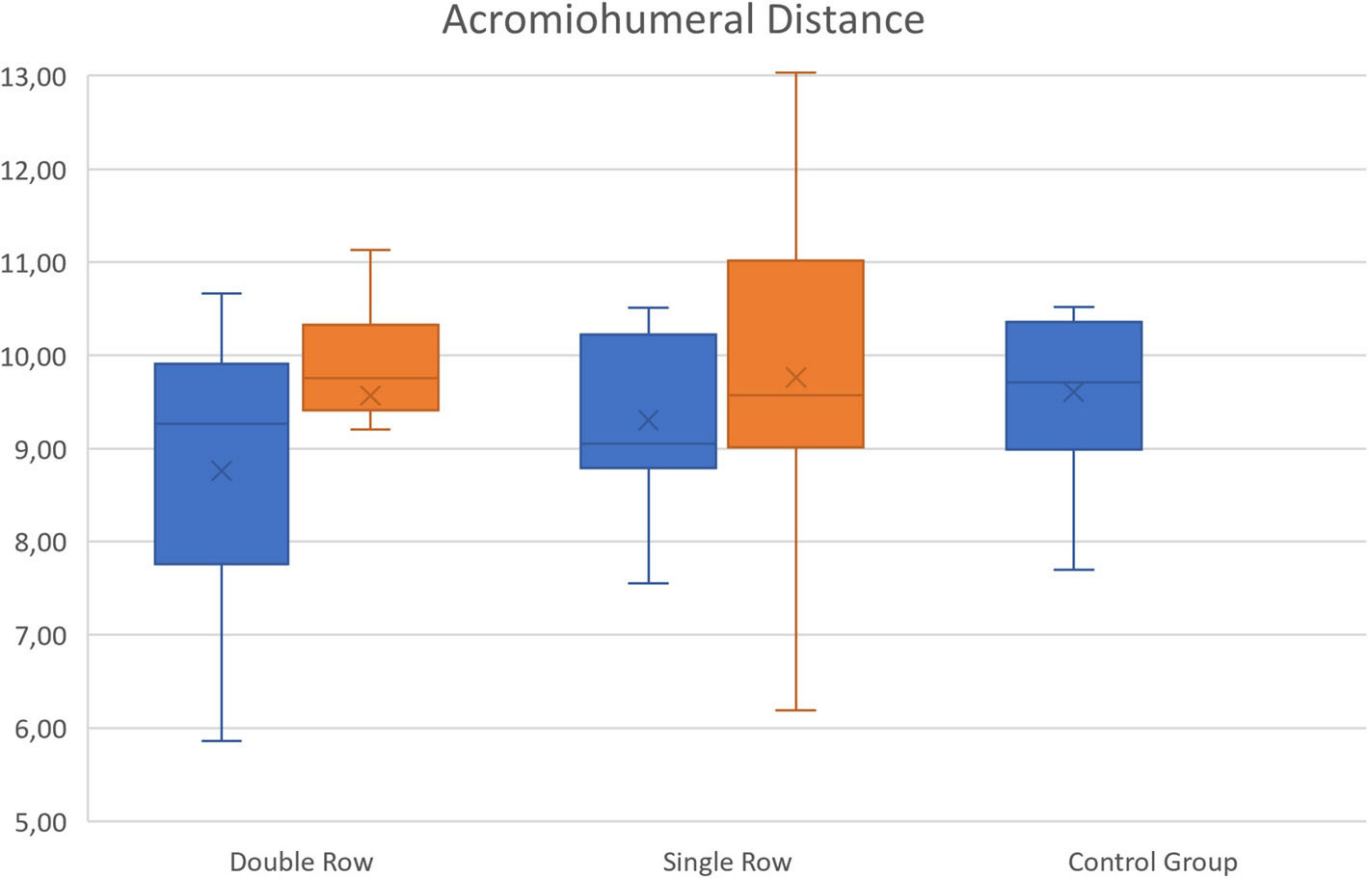
Chart of acromiohumeral distance analysis

**Fig. 4:**
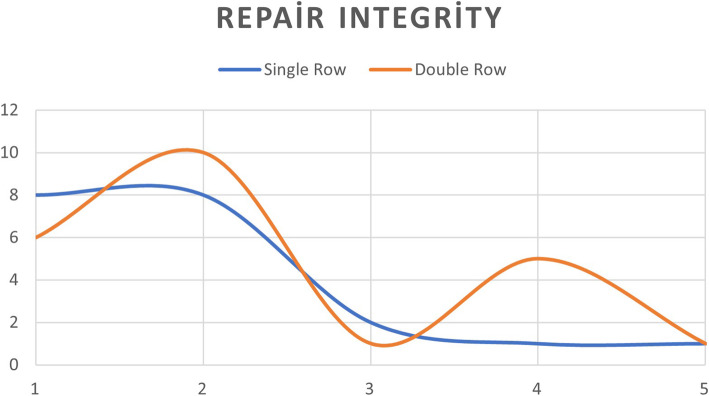
Chart of repair integrity assessment

The study included a total of 43 eligible patients, 34 females and 9 males. The mean age was 57.89 (45–78), and mean follow-up time was 28,65 (21–43) months (Tables 1 and 2). The mean AHD of the CG was 9.7 ± 0.96 mm, the preoperative AHD of DRG was 8.62 ± 1.45 mm, and SRG was 9.71 ± 0.95 mm. The postoperative mean AHD of DRG was 9.61 ± 1.83 mm, and SRG was 10.21 ± 1.97 mm (Table 2, Fig. 3). The mean size of tears was 10.81 ±4.03 mm. There were four tears with grade 1 and two with grade 2 fatty degeneration, and the rest with no fatty degeneration (grade 0). There was a total of 23 double-row and 20 single-row repairs. RI was determined type 1 for 14, type 2 for 18, type 3 for 3, type 4 for 6, and type 5 for 2 patients (Fig. 4). The inter-observer reliability was high (*ICC=0.876*) for the distance measurements and the RI classifications. AHDs of the preoperative and the control groups were not statistically different (*P=0.092*). On the other hand, the difference between the preoperative and postoperative group was significant (*P=0.009*). When the differences between the postoperative and control group distances were analyzed by repair method, the increase in the AHD in the double-row group was significantly higher than the single-row group (*P=0.004*). Good to excellent UCLA and qDASH scores were observed in 38 patients, and fair scores were observed in 5 patients; there was no statistical difference for the repair method on neither UCLA scores (*P=0.788*) nor qDASH scores (*P=0.578*). High correlation is assessed between the RI and qDASH scores (*P=0.005*), but no with UCLA scores (*P=0.399*) (Table 3). RI did not correlate with the repair method (*P*=0.580).

**Table 1:** Patient demographics

		Single-row	Double-row
Sex	Male	4	5
	Female	16	18
Dominant side	Right	18	19
	Left	2	4
Surgery side	Right	8	15
	Left	12	8
Mean age		54.95	60.83
Mean follow-up		29.90	27.57

**Table 2:** Measurements of acromiohumeral distance (AHD)

		AHD
Preoperative	DRG	8.62 ± 1.45
	SRG	9.71 ± 0.95
Postoperative	DRG	9.61 ± 1.83
	SRG	10.21 ± 1.97
Control group		9.70 ± 0.96

**Table 3:** Postoperative functional scores of the groups

	UCLA	P	qDASH	P
Single-row	31.55 ± 3.84	*.788*	4.65 ± 3.93	*.578*
Double-row	31.87 ± 2.07		3.85 ± 3.45	

## Discussion

Our study results show that the single- or double-row rotator cuff arthroscopic repairs do not significantly affect the RI. There are several studies for evaluating the superiority of the double-row repairs to the single-row repairs [1–3, 7, 15–17]. In some prospective randomized trials, Carbonel et al. and Ma et al. showed better functional results for double-row repairs on larger (>3cm) tears with no effect on RI [1, 18]. In contrast, Franceschi et al. and Papalia et al. recorded no clinical differences between the double- and single-row groups [19, 20]. Barber et al. have augmented both methods with platelet-rich plasma and fibrin membrane with no change in the clinical outcomes and RI [21]. We did not observe clinical findings specific to double-row repairs. Meyer et al. showed a low rate of tendon-to-bone healing in single-row repairs with an invasive imaging technique (MR arthrography), but a minimal influence on clinical outcome [22]. Especially for wider tears, double-row repairs may promise lower tear rate and better functionality, but this is still controversial [7].

In vitro studies showed superior biomechanical strength for double-row repairs and predicted better clinical results [8, 23–26]. Burkhart et al. showed a double-row technique to maximize the restoration of the anatomy [6]. Acan et al. evaluated the effect of lateral row anchor position on the strength of a double-row knotless repairs, demonstrating that the horizontal placement of the lateral anchor shows less cyclic elongation compared to more vertical placements [27]. We aim to place the lateral anchor horizontally when performing double-row repair. Longo et al. has also described a low profile knotless double-row repair technique to prevent the tear from catching below the acromion and avoid irritation of the subacromial space by knots [28]. The double-row repairs may restore the anatomy more than single-row repairs and lower the re-tear rate, but the clinical benefit is debatable [25, 29]. A study comparing the clinical effects of medial knotted and knotless double-row repairs at the end of the first year, found no difference in the rates of re-tear and clinical results between the knotted and knotless groups [30]. We preferred the medial knot method in the double-row repairs we performed.

AHD is the distance between humeral head and acromion. AHD is traditionally measured by conventional radiography, but inter- and intra-observer reliability is very low for this measurement method [31]; parameters like the position of the patient (standing or lying), x-ray beam angle, and rotation of the glenohumeral joint are known to interfere with the measurements [31–33]. Saupe et al. showed measurement with conventional radiography and MRI to be reliable regardless of observer experience, but the measurements from MRI are smaller than radiography [9]. Current evidence supports the reliability of ultrasound and CT or MR measurement [34]. Reduced AHD is associated with rotator cuff tear [34, 35], and some surgeons measure AHD for predicting the success of a rotator cuff tear repair [36]. Distances smaller than 7 mm are often associated with rotator cuff degenerative disease. The distance is not affected by acute traumatic tears in early stages.

Pepe et al. has done volume measurements with remodeling software, finding a significant subacromial volume increase in single-row repairs of degenerative full-thickness tears smaller than 3 cm [37]. They compared the distances with the contralateral shoulders as a healthy side control group. There is a high percentage of asymptomatic rotator cuff tears in the population, and patients undergoing rotator cuff repair have a higher prevalence of rotator cuff tear at the contralateral side [38], so contralateral shoulders cannot be assumed to be healthy. In our series, asymptomatic rotator cuff tear at the contralateral side was 53%. This ratio was consistent with the literature. We excluded the asymptomatic rotator cuff tears from the control group and compared the postoperative groups. Our hypothesis was that double-row repairs would preserve acromiohumeral range more than single-row repairs. Double-row repairs showed statistically more significant improvement on AHD, but this did not alter the outcome and clinical setting at the early and middle term. Nevertheless, we anticipate that gaining more AHD may prevent rotator cuff tear arthropathy seen at long-term follow-up.

The most significant limitation of our study is the size and heterogeneity of the groups. We have small and medium sized tears in the groups. If we include only medium sized tears, there may be better potential outcomes for double-row repair. Also, we did not obtain the control MRIs at the same postoperative date, which may have caused lower acromiohumeral distance measurements for late-control MRIs.

## Conclusion

Arthroscopic rotator cuff repairs have become the gold standard of treatment for most rotator cuff tears. Shorter hospital stays, less invasive technique, and faster rehabilitation are advantages of arthroscopic rotator cuff repairs, and the functional outcomes are promising. Regardless of the method, if we can achieve acceptable RI, then the functional results are favourable. Double-row repairs may provide better long-term outcomes due to preserving more acromiohumeral distance, but we still need more randomized controlled trials to definitively show the superiority of double repairs.

## Data Availability

This study does not contain any third materials.

## Abbreviations

ARCR: Arthroscopic rotator cuff repair
RI: Repair integrity
AHD: Acromiohumeral distance
DRG: Double-row group
SRG: Single-row group
CR: Control group
